# Clinical and epidemiological features of schistosomiasis among sub-Saharan African migrants in Barcelona, Spain: a retrospective observational study

**DOI:** 10.1186/s40249-026-01415-y

**Published:** 2026-02-05

**Authors:** Derlis Duarte-Zoilan, Fernando Salvador, Cristina Bocanegra, Elena Sulleiro, Adrián Sánchez-Montalvá, Núria Serre-Delcor, Pau Bosch-Nicolau, María Luisa Aznar, Lidia Goterris, Diana Pou, María Espiau, Patricia Martínez-Vallejo, Joan Martínez-Campreciós, Juan Espinosa-Pereiro, Inés Oliveira, Francesc Zarzuela, Israel Molina

**Affiliations:** 1 Department of Medicine, Autonomous University of Barcelona, Barcelona, Spain; 2 Department of Infectious Diseases, Unit of International Health Vall d’Hebron-Drassanes, Vall d’Hebron University Hospital, PROSICS Barcelona, Pº Vall d’Hebron 119-129, 08035 Barcelona, Spain; 3 Biomedical Research Networking Center for Infectious Diseases (CIBERINFEC), Carlos III Health Institute, Madrid, Spain; 4 Department of Microbiology, Vall d’Hebron University Hospital, PROSICS Barcelona, Barcelona, Spain; 5 Unit of Pediatric Infectious Diseases and Immunodeficiencies, Vall d’Hebron University Hospital, PROSICS Barcelona, Barcelona, Spain

**Keywords:** Schistosomiasis, Migrant, Sub-Saharan Africa, Serology, Praziquantel

## Abstract

Schistosomiasis is a neglected tropical disease, affecting 253 million people worldwide. This study aimed to describe the clinical and epidemiological characteristics of schistosomiasis in sub-Saharan African migrants evaluated through a general screening program for imported infectious diseases at a specialized international health unit in Barcelona, Spain. We performed a retrospective observational study (2014–2023) including confirmed (defined by egg detection in stool or urine) and probable cases (identified by positive serology against *Schistosoma)*. Data on epidemiology, clinical presentation, diagnosis, treatment, and follow-up were collected. Among 3214 screened migrants, 855 (26.6%) were diagnosed with schistosomiasis. Most were male (87%) with a median age of 21.9 years, mainly originating from Mali, Gambia, and Senegal. Only 2.2% reported symptoms, while anemia and eosinophilia were observed in 9.7% and 29.7% of cases, respectively. Parasitological confirmation was achieved in 24.9%, with the remainder diagnosed by serology alone. Abdominal ultrasound was performed in 122 patients, revealing abnormalities in 20.4%. Multivariate analysis showed that confirmed cases tended to be younger (22.4 years vs 25.8 years) and had higher frequencies of women (17.8% vs 11.3%), symptoms (5.6% vs 1.1%), eosinophilia (49.4% vs 22.5%), and ultrasound abnormalities (22.7% vs 17.8%) than probable cases. Praziquantel treatment was administered to 775 patients (90.6%). Follow-up information was available for 218 individuals (28.1%); among those, eggs persisted in 7.4% (9/122), and 84% (74/88) maintained positive serology. Schistosomiasis prevalence in this migrant population was high and mostly asymptomatic, with limited laboratory abnormalities but notable radiological findings, particularly in confirmed cases. Serology was useful diagnostically but had limited value in follow-up. Systematic screening of at-risk populations remains crucial for early detection and treatment.

**Graphical Abstract:**

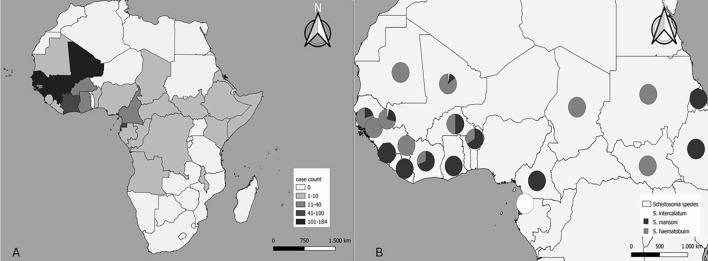

## Background

Schistosomiasis is a long-lasting and debilitating parasitic disease caused by trematodes of the genus *Schistosoma*. The World Health Organization (WHO) reported that 253 million people were affected by schistosomiasis in 2024, with more than 90% of them living in sub-Saharan Africa [1, 2]. It constitutes a major public health problem, being included by the WHO in the list of neglected tropical diseases, and it is present in 78 countries across four continents [3, 4]. Transmission occurs through contact with freshwater contaminated with cercariae released by freshwater snails, which act as intermediate hosts. The primary activities related to infection are routine agricultural, domestic, occupational, and recreational activities [3].

In Spain, schistosomiasis is an imported disease, primarily observed in migrants and travelers from endemic areas, mostly from sub-Saharan Africa, where *Schistosoma haematobium*, *S. mansoni, and S. intercalatum/S. guineensis* are the predominant species [5]. The increase in international mobility and migration flows has led to a rise in the number of cases detected in non-endemic countries. However, recent reports of potential autochthonous transmission (as observed in Corsica and Almeria) have raised new concerns, posing additional challenges for the diagnosis and clinical management of the disease [6–8]. Chronic infection related to schistosomiasis is more common among individuals living in endemic areas where repeated or continuous exposure to the parasite is common. Recently arrived migrants have similar manifestations as people infected in endemic areas [9, 10].

The severity of the cases is related to the amount of eggs trapped in the tissues, their anatomical distribution, the duration and intensity of the exposure, and the host’s immune response, with symptoms presenting insidiously in most cases [11–13]. In the most severe cases, inflammation in the intestines can lead to ulceration and blood loss, while in the liver, periportal fibrosis (Symmer's stem fibrosis), portal hypertension, and collateral circulation may occur. In the urinary tract, the development of granulomatous inflammation of the bladder may cause wall thickening with pseudopolyps and/or obstruction, potentially progressing to kidney failure. Persistent schistosomiasis has been associated with an increased risk of bladder cancer [14–16]. Female genital schistosomiasis is characterized by the deposition of eggs in the tissues of the genital tract (including the cervix, vagina, and vulva) which leads to chronic granulomatous lesions associated with infertility, dyspareunia, vaginal bleeding, and an increased susceptibility to sexually transmitted infections, particularly human immunodeficiency virus (HIV), with a relative risk up to three times higher in affected women [17].

The diagnosis of schistosomiasis in migrants poses a challenge due to variability in the clinical presentation. Additionally, the lack of awareness among healthcare professionals in non-endemic countries can lead to underdiagnosis or delayed diagnosis, which increases the risk of complications. These diagnostic barriers highlight the need for proactive, systematic screening of in migrant populations. Screening for infectious diseases in migrants is a fundamental public health strategy, as it allows early detection of conditions prevalent in their countries of origin (often asymptomatic or presenting with nonspecific symptoms) such as tuberculosis infection, viral hepatitis, HIV, and parasitic infections like schistosomiasis. These diseases can persist undetected for years without an active search, leading to serious complications and increasing the risk of transmission [18, 19].

This study describes the demographics, clinical features, laboratory and microbiological findings, treatment, and follow-up of schistosomiasis cases detected through screening among sub-Saharan African migrants attending a referral unit in a non-endemic setting.

## Methods

### Study population and protocol.

We conducted a retrospective observational study including migrants from sub-Saharan Africa who were screened and diagnosed with schistosomiasis at the International Health Unit Vall d’Hebron–Drassanes (Barcelona, Spain) between January 2014 and December 2023. In this study, ‘migrant’ is defined according to Eurostat as a person who has moved from their country of origin to another country with the intention of residing there temporarily or permanently [20]. The International Health Unit offers a structured health status screening to newly arrived migrants in order to early detect and treat prevalent infectious diseases. In migrants from sub-Saharan Africa, schistosomiasis screening included the following assessments: a general blood test (including blood cell count and biochemistry), microscopic examination of one sample of feces (through the formalin-ether technique of Ritchie) and one sample of urine (after centrifugation), specific serological technique for detecting *a Schistosoma IgG ELISA* (Novagnost *S. mansoni* IgG; Siemens Diagnostics, Marburg, Germany*).* Abdominal ultrasound was not routinely performed but ordered at the treating physician’s discretion. No specific screening for female genital schistosomiasis was performed routinely. The diagnosis of schistosomiasis was classified as confirmed (defined by the presence of *Schistosoma* eggs in stool or urine), or probable (defined as having a positive specific *Schistosoma* serology in the absence of egg detection). In addition to schistosomiasis screening, patients underwent a systematic evaluation for co-infections, including an interferon-gamma release assay (IGRA) for tuberculosis infection and a chest X-ray; serological testing for hepatitis B and C virus, HIV, *Strongyloides stercoralis*, and syphilis; a polymerase chain reaction (PCR) to detect different species of *Plasmodium*; and a microfilariae detection in peripheral blood through the technique based on a leuko-concentration with saponin.

The following variables were extracted from medical records: demographic data (sex, age, country of birth, and duration of residence), symptoms, immunosuppressive conditions, co-infections, laboratory findings (eosinophilia and hemoglobin level), radiological tests, microbiological results (serology and stool/urine microscopy), treatment received, and clinical and microbiological follow-up. Eosinophilia was defined as an absolute eosinophil count of ≥ 500 cells/μl (using the ≥ 500 cells/μl definition ensures high specificity for clinically meaningful eosinophilic responses in helminth infections), and anemia as a hemoglobin level ≤ 120 g/L in women and ≤ 130 g/L in men. According to our unit’s follow-up protocol, patients with confirmed schistosomiasis underwent a stool/urine microscopic examination at least one month after treatment to evaluate egg elimination; when eosinophilia was present at baseline, a control eosinophil count was performed 3–4 months post-treatment; a control serological test was performed 6 months after treatment. A control abdominal ultrasound was performed in those presenting baseline alterations 6 months after treatment.

### Statistical analysis

The database was designed in Microsoft Excel (Office2016, Microsoft, Redmond, USA) and once completed, was transferred to the Epi Info™ free software for Windows (version 7.2.5.0, CDC, USA) for analysis. QGIS free software for Windows (version 3.28.0, available from https://qgis.org/downloads-list/) was used for map creation. The Shapiro-Wilk test was performed to determine the normality of the distribution of the variables. Qualitative variables were expressed as absolute numbers and percentages, while quantitative variables were expressed as mean and standard deviations (SD) or median and interquartile range (IQR). Categorical variables were compared using the chi-squared test or the Fisher test when appropriate. Continuous variables were compared using the Student’s *t*-test or the Kruskal-Wallis test. Variables showing statistical significance in the univariate analysis (*P* < 0.05) were included in a multivariate logistic regression model to determine adjusted odds ratios (a*OR*) and identify independent associations with confirmed diagnosis. To assess the evolution of serological markers and eosinophilia, a regression analysis was conducted using linear mixed-effects models. The models were estimated via maximum likelihood using an Expectation–Maximization optimization algorithm, and the statistical significance of the coefficients was assessed using Wald tests. These analysis were performed using Stata v17.0 (StataCorp LLC, Texas, USA).

## Results

### Epidemiological and clinical characteristics

**Fig. 1:**
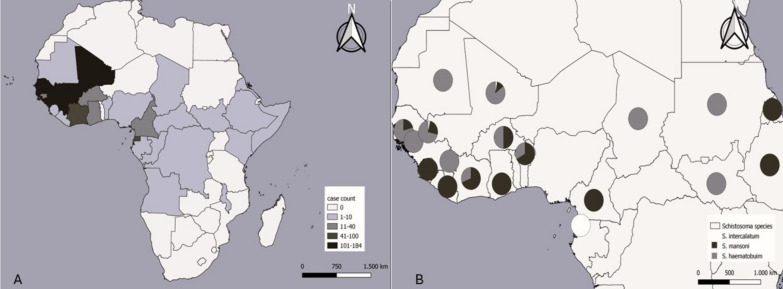
Geographical distribution of schistosomiasis cases (**A**) and distribution of *Schistosoma* species in confirmed cases (**B**)

During the study period, 3214 migrants from 27 sub-Saharan African countries were screened, and eight hundred fifty-five (26.6%) participants were diagnosed with schistosomiasis. They were predominantly male (744 cases, 87.0%), with a median age of 22 (IQR: 17–28) years. The countries with the highest number of cases were concentrated in the West African region, mainly in Mali (184 cases), Gambia (155 cases) and Senegal (148 cases), and median time of residence in our country was 4.4 (IQR: 2.0–10.1) months. The number of cases and percentages for each country are shown in Table 1 and Fig. 1A.

**Table 1:** Distribution of schistosomiasis cases by country of origin at the Tropical Medicine Referral Unit, 2014–2023

Countries of origin	Number of cases (percentage, %)
Mali	184/333 (55.3)
Gambia	155/544 (28.5)
Senegal	148/672 (22.0)
Guinea Conakry	109/306 (35.6)
Equatorial Guinea	81/525 (15.4)
Ivory Coast	48/151 (31.8)
Ghana	32/155 (20.6)
Cameroon	14/111 (12.6)
Burkina Faso	12/41 (29.3)
Guinea-Bissau	12/51 (23.5)
Nigeria	10/121 (8.3)
Sierra Leone	10/23 (43.5)
Sudan	7/10 (70.0)
Benin	5/9 (55.6)
Angola	4/15 (26.7)
Somalia	4/34 (11.8)
Congo	3/12 (25.0)
Ethiopia	3/6 (50.0)
Gabon	3/4 (75.0)
Mauritania	3/11 (27.3)
Liberia	2/6 (33.3)
Chad	1/4 (25.0)
Eritrea	1/3 (33.3)
Kenya	1/12 (8.3)
Malawi	1/1 (100.0)
Democratic Republic of the Congo	1/10 (10.0)
Central African Republic	1/14 (7.1)
Total	855/3214 (26.6)

Only 19 (2.2%) of the cases presented any symptom related to schistosomiasis. The most frequent symptoms were hematuria in 16 (1.8%), dysuria in 14 (1.6%), and abdominal pain in 2 (0.2%). Regarding laboratory tests, eosinophilia was present in 248 (29.7%) patients, and anemia in 81 (9.7%) of the patients.

In the overall cohort, 213 out of 855 (24.9%) migrants had confirmed schistosomiasis. Urine samples were collected from 831 migrants, and stool samples from 828. Of these, 96 (11.5%) and 122 (14.7%) were positive. The remaining 642 (75.1%) migrants had a positive serology in the absence of egg detection, and were considered probable cases. Only 9 cases had a confirmed diagnosis through microscopy but a negative serology. Of the confirmed cases, the following species were identified: 91 (42.7%) were *S. haematobium*, 71 (33.3%) were *S. mansoni*, 46 (21.6%) were *S. intercalatum/S. guineensis*, and co-infection was observed in 5 (2.3%) cases (see Table 2). The geographical distribution of confirmed cases was different depending on the *Schistosoma* species (see Fig. 1B).

**Table 2:** Epidemiological and clinical characteristics of sub-Saharan African migrant patients with schistosomiasis attended at the Tropical Medicine Referral Unit, 2014–2023

Epidemiological and clinical characteristics	Number of patients (*n* = 855)
Sex, male	744/855 (87.0)
Median age (years)	22 (IQR: 17–28)
Median time of residence in Spain (months)	4.4 (IQR: 2–10.1)
*Clinical symptoms*	
Presence of any symptom	19/855 (2.2)
Hematuria	16/855 (1.8)
Dysuria	14/855 (1.6)
Abdominal pain	2/855 (0.2)
*Laboratory abnormalities*	
Anemia	81/835 (9.7)
Eosinophilia	248/835 (29.7)
*Diagnosis*	
Probable cases	642/855 (75.1)
Confirmed cases	213/855 (24.9)
*Microbiological tests*	
Positive serological test	763/772 (98.8)
Positive urine microscopic examination	96/831 (11.5)
Positive stool microscopic examination	122/828 (14.7)
*Schistosoma species in confirmed cases*	
*S. haematobium*	91/213 (42.7)
*S. mansoni*	71/213 (33.3)
*S. intercalatum/S. guineensis*	46/213 (21.6)
*S. haematobium* + S. *mansoni*	4/213 (1.8)
*S. haematobium* + *S. intercalatum/S. guineensis*	1/213 (0.4)
*Ultrasound findings*	
Abnormal findings related to schistosomiasis	25/122 (20.4)
Bladder wall thickening	11/122 (9.0)
Bladder calcifications	5/122 (4.1)
Hepatomegaly	3/122(2.4)
Hydronephrosis	3/122 (2.4)
Splenomegaly	2/122 (1.6)
Portal hypertension	2/122 (1.6)
Liver fibrosis	1/122 (0.8)

Data are reported as number (%) of patients

Overall, 122 (14.2%) abdominal ultrasounds were performed. Of these, radiological alterations were identified in 25 cases (20.4%). The most frequent observed findings were: bladder wall thickening in 11 patients (9.0%), bladder calcifications in 5 (4.1%), hepatomegaly in 3 (2.4%), and hydronephrosis in 3 patients (2.4%) (see Table 2).

Main co-infections diagnosed among this cohort of patients were: tuberculosis infection (186 cases, 21.7%), chronic hepatitis B virus infection (86, 10.0%), malaria (80, 9.3%), strongyloidiasis (48, 5.6%), giardiasis (24, 2.8%), *Trichuris trichiura* infection (22, 2.6%), *Mansonella* sp. infection (16, 1.8%), hookworms infection (9, 1%), *Ascaris lumbricoides* infection (8, 0.9%), HIV infection (8, 0.9%), syphilis (7, 0.8%), *Entamoeba histolytica* infection (4, 0.4%), *Loa loa* infection (3, 0.3%), chronic hepatitis C virus infection (3, 0.3%), pulmonary tuberculosis disease (2, 0.2%).

### Comparisons among different groups

In the comparison of clinical and epidemiological characteristics between confirmed and probable cases, the univariate analysis showed significant differences. Confirmed cases included a higher proportion of women (17.8% vs 11.3%, *P* = 0.020), symptomatic patients (17.8% vs 1.1%, *P* < 0.001), individuals with eosinophilia (49.4% vs 22.5%, *P* < 0.001), and ultrasound abnormalities (22.7% vs 17.8%, *P* < 0.001). In addition, confirmed cases had a lower median age (22.4 vs 25.8 years, *P* < 0.001) and a shorter median duration of residence in the country (3.3 vs 4.8 months, *P* < 0.001). These associations were confirmed in the multivariate analysis (see Table 3).

**Table 3:** Comparison of clinical, laboratorial, and radiological characteristics between confirmed and probable cases of sub-Saharan African migrant patients with schistosomiasis attended at the Tropical Medicine Referral Unit. 2014–2023

Univariate analysis				Multivariate analysis	
Clinical and epidemiological characteristics	Confirmed (*n* = 213)	Probable (*n* = 642)	*P*-value	Adjusted odds ratio (*aOR*) (95% *CI*)	*P*-value
Sex, female	38/213 (17.8%)	73/642 (11.3%)	0.020	2.6 (1.5–4.6)	0.001
Median age, years	22.4 (IQR = 20.2–24.2)	25.8 (IQR: 23.5–26.9)	< 0.001	0.9 (0.9–0.9)	< 0.001
Median time of residence in Spain, months	3.3 (IQR = 1.5–8.1)	4.8 (IQR: 2.1–11.3)	< 0.001	1.0 (0.9–1.1)	0.004
Presence of symptoms	12/213 (5.6%)	7/642 (1.1%)	< 0.001	5.3 (1.9–14.8)	< 0.001
Presence of eosinophilia	104/210 (49.4%)	144/639 (22.5%)	< 0.001	3.4 (2.4–4.7)	< 0.001
Presence of anemia	22/210 (10.4%)	59/639 (9.2%)	0.721	–	–
Presence of ultrasound abnormalities	15/66 (22.7%)	10/56 (17.8%)	< 0.001	4.9 (1.9–12.1)	0.001

Data are reported as number (%) of patients, or median (IQR). The multivariate model was adjusted for the following variables, selected as potential confounding factors based on their clinical and epidemiological relevance: sex, age, duration of residence in Spain, presence of symptoms, eosinophilia, and abnormal ultrasound findings

Baseline characteristics differed significantly among individuals with confirmed infection according to *Schistosoma* species. The proportion of female participants was significantly higher in the *S. intercalatum/S. guineensis* group (67.3%). Median age also showed significant differences, with *S. intercalatum/S. guineensis* cases being older (24.1 years) compared to the other two groups (20.0% and 18.9%). The presence of symptoms was more frequent in *S. haematobium* group (10.9%) than in *S. intercalatum* (2.1%) and *S. mansoni* (1.4%). Positive serology was more common in *S. mansoni* (97.7%) and *S. haematobium* (93.1%) groups than in *S. intercalatum/S. guineensis* (78.5%). Anemia was notably more prevalent in the *S. intercalatum/S. guineensis* group (32.6%) (see Table 4).

**Table 4:** Comparison of clinical and epidemiological characteristics among patients infected with different *Schistosoma* species in confirmed cases of sub-Saharan African migrant patients with schistosomiasis attended at the Tropical Medicine Referral Unit. 2014–2023

Clinical and epidemiological characteristics	*S. intercalatum/S. guineensis*	*S. mansoni*	*S. haematobium*	*P*-value
Sex, female	31/46 (67.3)	6/71 (8.4)	1/91 (1)	< 0.001
Median age, years	24.1 (IQR: 24.1–27.4)	20.1 (IQR: 17.6–25.1)	18.9 (IQR: 17.9–24.0)	< 0.001
Presence of symptoms	1/46 (2.1)	1/71 (1.4)	10/91 (10.9)	0.027
Presence of eosinophilia	22/45 (48.8)	36/67 (53.7)	47/88 (53.4)	0.800
Positive serology	11/14 (78.5)	44/45 (97.7)	68/73 (93.1)	0.047
Presence of anemia	15/46 (32.6)	8/68 (11.7)	2/89 (2.2)	< 0.001
Ultrasound abnormalities	1/4 (25.0)	4/14 (28.5)	10/46 (21.7)	0.821

Data are reported as number (%) of patients or median (IQR)

## Treatment information and follow-up

**Fig. 2:**
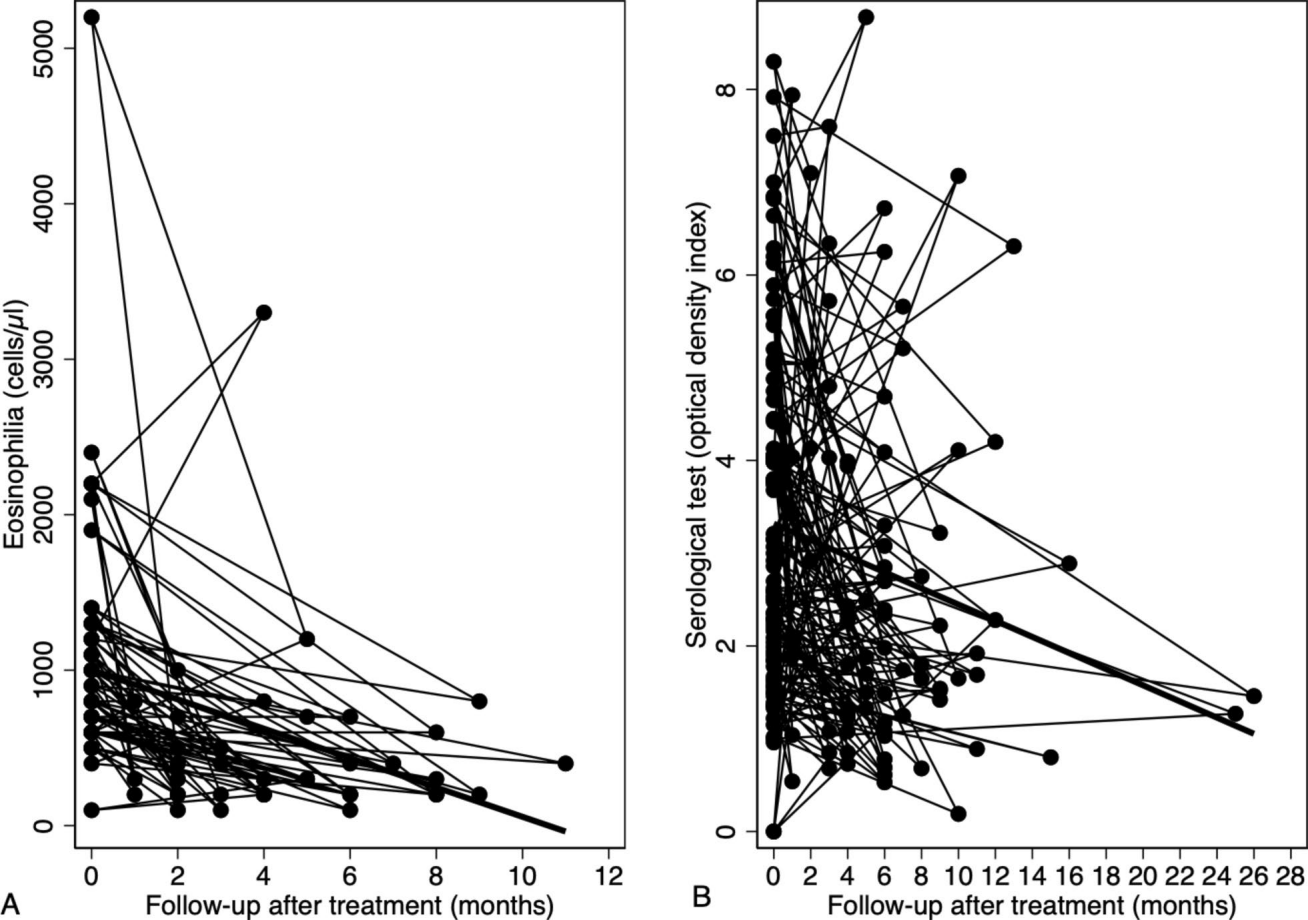
Evolution of eosinophilia (**A**) and serology (**B**) after treatment in sub-Saharan African migrant patients with schistosomiasis treated at the Tropical Medicine Reference Unit. 2014–2023. Thin lines represent individual trend in the values, while thick lines represent the overall trend measured through mixed-effects regression models

Treatment was given to 755 (90.6%) patients, while 9.3% (80 patients) did not receive any treatment because they were lost to follow-up early after the diagnosis was established. Regarding praziquantel treatment regimens, a clear preference was observed for the 40 mg/kg for two days regimen, which was given to 544 patients (70.1%). Another frequent regimen used was 40 mg/kg for one day, administered to 227 patients (29.2%) (see Table 5). Follow-up information was available for 218 (28.1%) of the patients. Only 122 patients with confirmed diagnosis had a microscopic stool/urine examination during follow-up with a median time of post-treatment evaluation of 2 (IQR: 1–3) months, of which 1/62 (1.6%) tested positive in stool and 8/60 (13.3%) tested positive in urine. In most of these cases, non-viable eggs were observed or the study was performed before 30 days after treatment. Regarding patients with baseline eosinophilia, 13/43 (30.2%) cases showed a sustained elevated eosinophil count with a median time of post-treatment evaluation of 2 (IQR: 1–6) months. Of the 25 cases with ultrasonographic abnormalities, ultrasonography was performed after treatment in 23 (92.0%) cases: radiological abnormalities persisted in 12 (52.2%) cases, while improvement or resolution of abnormalities was observed in 2 (8.7%) and 9 (39.1%) cases respectively. As for serological follow-up, it was possible to perform it in 88 patients, and 74 (84.1%) of these cases remained positive with a median time of post-treatment evaluation of 6 (IQR: 4–12) months. Analysis using mixed-effects regression models revealed a significant association between serology and eosinophilia in relation to the time variable after treatment. For serology (see Fig. 2A), the model showed a mean decrease in the optical density index of 0.078 (*P* = 0.001), along with significant variance in the slope (SD = 0.0859). On the other hand, the eosinophilia model (see Fig. 2B) demonstrated a mean decrease of 104.6 cells/μl (*P* < 0.0001), with negligible variance, suggesting a more uniform temporal pattern among individuals.

**Table 5:** Treatment received and clinical outcomes after treatment in sub-Saharan African migrant patients with schistosomiasis attended at the Tropical Medicine Referral Unit. 2014–2023

Treatment and clinical outcomes	Number of patients (*n* = 855)
Patients receiving treatment	775/855 (90.6)
*Therapeutic regimens with praziquantel*	
40 mg/kg for one day	227/775(29.2)
40 mg/kg for two days	544/775 (70.1)
40 mg/kg for three days	4/755 (0.5)
*Follow-up information*	
Patients with follow-up information	218/775 (28.1)
Positive stool microscopic examination during follow-up	1/62 (1.6)
Positive urine microscopic examination during follow-up	8/60 (13.3)
Post-treatment eosinophilia	13/43 (30.2)
Positive serology after treatment	74/88 (84.0)
Post-treatment ultrasound abnormalities	12/23 (52.2)

Data are reported as number (%) of patients

## Discussion

The findings of this study reveal a high prevalence of schistosomiasis (26.6%) among migrant individuals from sub-Saharan Africa, particularly from West Africa, with a higher representation of patients coming from Mali, Gambia, and Senegal. These data are consistent with previous studies identifying this region as hyperendemic for *S. haematobium* and *S. mansoni*, due to environmental and socioeconomic factors that facilitate transmission [3, 18]. Despite the high number of cases in our cohort, most individuals were asymptomatic at presentation, a pattern well documented in the literature for chronic or low-parasite-load infections [21]. This finding underscores the need for systematic screening in migrant populations, even in the absence of symptoms, as recommended by various international guidelines [22].

The diagnostic methods employed showed that only one-quarter of cases were confirmed by direct methods (egg detection in urine or stool), reflecting the limited sensitivity of these techniques in low-parasite-load contexts [23]. In contrast, serology enabled the detection of a large proportion of probable cases, confirming its utility as a diagnostic tool in non-endemic settings [22, 24]. However, it would be pertinent to consider as a significant limitation the fact that serology for *Schistosoma* does not distinguish between active and past infections. In this context, it is reasonable to assume that a substantial number of subjects classified as probable schistosomiasis cases may actually correspond to individuals with previously resolved infections. This could distort the interpretation of the sensitivity of direct methods and the true diagnostic value of serology in this group. Moreover, the persistence of serological positivity during follow-up (84%) also poses challenges in post-treatment interpretation, as although a small decrease in serological levels was observed, the vast majority remain positive after treatment, precluding the use of this technique as early biomarker of cure [25]. Therefore, more prospective studies are needed to identify reliable tools for assessing treatment response and confirming parasitological cure.

These values must be interpreted with caution, as they may also be influenced by co-infections with other diseases, including other parasitic infections. From a clinical perspective, eosinophilia and anemia were common findings, the latter being significantly more prevalent in *S. intercalatum* infections. This pattern has been previously described and may reflect distinct immunopathological mechanisms among species [26, 27]. The significant association between baseline eosinophilia and confirmed cases supports its use as an ancillary biomarker for diagnostic suspicion [24, 28]. Regarding imaging, the most frequent ultrasound findings were bladder wall thickening and the presence of bladder calcifications, typical features of chronic urogenital schistosomiasis. Although these alterations were more prevalent among confirmed cases, their presence in probable cases suggests that alterations may arise from enhanced immune response partially independent from the disease burden [18].

Most commonly used regimens (40 mg/kg for one or two days) was consistent with evidence suggesting greater efficacy against mixed or intense infections [29]. However, the limited follow-up information (only 28.1% of the treated patients) and the persistence of eosinophilia in one-third of patients highlight the need for more robust and long-term follow-up strategies. An additional treatment course could be considered in patients with minimal reduction in the eosinophilia or serologic levels [30, 31]. Additional reason for eosinophilia should be ruled out before offering an additional new course of treatment. Regression models showed a significant reduction in eosinophilia and serology over time, although with significant inter-individual variability in serological response. This finding raises the possibility of developing strategies for post-treatment monitoring, particularly relevant in mobile populations such as migrants [32].

The strong association between eosinophilia and confirmed cases observed in our study is consistent with previous research highlighting eosinophilia as a hallmark of helminthic infections, particularly in the acute or tissue-invasive phases of schistosomiasis [12, 21]. Eosinophil counts often reflect host immune response to parasite antigens and can serve as a useful screening marker in endemic and migrant populations, although their specificity remains limited [33]. The observed relationship reinforces the importance of integrating routine hematological assessments in the diagnostic approach for suspected parasitic infections, especially when access to parasitological or serological confirmation is restricted.

Moreover, women accounted for 67.3% of cases with *S. intercalatum*/*S. guineensis* infection, in contrast to only 8.4% and 1% in cases with *S. mansoni* and *S. haematobium*, respectively. The reason of these difference could be explained because all the cases of *S. intercalatum*/*S. guineensis* came from Equatorial Guinea; the pattern of migration from this country to Spain is different from the pattern of migration from other sub-Saharan countries (higher proportion of women in migrants coming from Equatorial Guinea compared with other countries of West and Central Africa). In addition, the type of exposure (use of water in domestic tasks, for example) may be mediated by gender roles [34].

This study has limitations that should be acknowledged. First, it is a retrospective study, and data were collected from electronic medical records, which may have resulted in missing information. Second, most cases were classified as probable, with diagnosis based on serological positivity, and some false positives may have been included in the study. Third, radiological test were not performed routinely (requested under physician criteria, most probably when patients had symptoms or eggs were detected), as well as female genital schistosomiasis screening, and the information could be biased. Finally, due to the high mobility of this population, information regarding the post-treatment follow-up is scarce.

## Conclusions

In summary, the prevalence of schistosomiasis among migrants coming from sub-Saharan Africa to our Unit was high, particularly among those coming from West Africa, although most of them were asymptomatic. Even though egg detection is the confirmatory diagnostic methods, most of the patients were diagnosed through serological testing. These results reinforce the importance of considering schistosomiasis as a significant public health concern in the context of migrants from endemic areas. The implementation of screening strategies, serological diagnosis, treatment, and personalized follow-up emerges as a priority to reduce the morbidity associated with this neglected infection in host countries.

## Data Availability

Data that support the findings of this study are available from the corresponding author upon reasonable request.

## Abbreviations

WHO: World Health Organization
HIV: Human Immunodeficiency Virus
IGRA: Interferon-gamma release assay
PCR: Polymerase chain reaction
SD: Standard deviations
IQR: Interquartile range
a*OR*: Adjusted odds ratios
